# Potential Determinants for Radiation-Induced Lymphopenia in Patients With Breast Cancer Using Interpretable Machine Learning Approach

**DOI:** 10.3389/fimmu.2022.768811

**Published:** 2022-06-21

**Authors:** Hao Yu, Fang Chen, Ka-On Lam, Li Yang, Yang Wang, Jian-Yue Jin, Aya EI Helali, Feng-Ming (Spring) Kong

**Affiliations:** ^1^ Institute of Biomedical and Health Engineering, Chinese Academy of Sciences Shenzhen Institutes of Advanced Technology, Shenzhen, China; ^2^ Department of Clinical Oncology, University of Hong Kong-Shenzhen Hospital, Shenzhen, China; ^3^ Department of Clinical Oncology, The University of Hong Kong, Hong Kong, Hong Kong SAR, China; ^4^ Biomedical Engineering, Shenzhen Polytechnic, Shenzhen, China; ^5^ University Hospitals/Cleverland Medical Center, Seidman Cancer Center and Case Western Reserve University, Cleveland, OH, United States

**Keywords:** radiation-induced lymphopenia, breast cancer, radiation dose, machine learning, SHapley Additive exPlanation

## Abstract

Radiation-induced lymphopenia is known for its survival significance in patients with breast cancer treated with radiation therapy. This study aimed to evaluate the impact of radiotherapy on lymphocytes by applying machine learning strategies. We used Extreme Gradient Boosting (XGboost) to predict the event of lymphopenia (grade≥1) and conduced an independent validation. Then, we induced feature attribution analysis (Shapley additive explanation, SHAP) in explaining the XGboost models to explore the directional contribution of each feature to lymphopenia. Finally, we implemented the proof-of-concept clinical validation. The results showed that the XGboost models had rigorous generalization performances (accuracies 0.764 and ROC-AUC 0.841, respectively) in the independent cohort. The baseline lymphocyte counts are the most protective feature (SHAP = 5.226, direction of SHAP = -0.964). Baseline platelets and monocytes also played important protective roles. The usage of taxane only chemotherapy was less risk on lymphopenia than the combination of anthracycline and taxane. By the contribution analysis of dose, we identified that firstly lymphocytes were sensitive to a radiation dose less than 4Gy; secondly the irradiation volume was more important in promoting lymphopenia than the irradiation dose; thirdly the irradiation dose promoted the event of lymphopenia when the irradiation volume was fixed. Overall, our findings paved the way to clarifying the radiation dose volume effect. To avoid radiation-induced lymphopenia, irradiation volume should be kept to a minimum during the planning process, as long as the target coverage is not compromised.

## Introduction

The biological effects of radiation exposure on the immune system are double-edged. It has an immunostimulatory effect by promoting the release of tumor antigens (1), radiation-induced neoantigens (2) and chemokine that recruit effector cells into the tumor microenvironment (3). On the other side, radiation has the potential for direct cytotoxicity toward immune cells, especially lymphocytes, which are the most radiosensitive (4). Among human peripheral blood lymphocytes, T helper cells, cytotoxic T cells, and B cells display a radiosensitive phenotype (5). In the treatment of solid tumors, lymphopenia is a common side effect of radiotherapy known for decades (6).

Radiation-induced lymphopenia is associated with inferior clinical outcomes in a wide variety of solid malignancies (7, 8), and more importantly, it is associated with inferior survival outcomes (9–12). For example, total lymphocyte counts < 100 cells/mm^3^ were associated with poor overall survival in patients with locally advanced cervical cancer (13); total lymphocyte count < 500 cells/mm^3^ at 2 months was associated with short overall survival outcomes and was an independent predictor for survival in elderly patients with glioblastoma (10). Similarly, lymphopenia was an independent predictor of inferior survival in locally advanced pancreatic cancer (11). In patients with breast cancer, the five-year disease-free survival was significantly lower in patients with a ratio of lymphocyte nadir to pre-treatment lymphocyte less than 0.8 (14). Nevertheless, baseline characteristics associated with radiation-induced lymphopenia have not been thoroughly evaluated. Given its clinical implications, it is necessary to identify those baseline characteristics to predict radiation-induced lymphopenia.

Recently, some studies have investigated and modeled the significant effects of radiation dose on radiation-induced lymphopenia. In esophageal cancer patients who underwent radiotherapy, thoracic vertebral (TV) volume spared of 5-40Gy was significantly associated with higher lymphocyte nadirs (P<0.05) (15). Yovino et al. (16) demonstrated the lymphotoxic impact of conventionally fractionated brain radiotherapy for high-grade gliomas. They established that after 30 fractions of radiotherapy, 99% of the circulating lymphocyte received ≥0.5Gy. Furthermore, our group identified a model of the effective radiation dose to the circulating immune cells (EDIC) in patients with advanced esophageal squamous cell carcinoma treated with trimodality therapy (17, 18). EDIC negatively correlated with lymphocyte nadir. Overall, radiation therapy was associated with lymphopenia in patients with different solid tumors. However, the association between breast cancer and radiation-induced lymphopenia is less well-studied.

In this study, we generated Extreme Gradient Boosting (XGboost) (19) models in a machine learning framework to identify the impact of radiation dose on circulating lymphocytes in patients with breast cancer. XGboost is the specific implementation of the gradient boosting algorithm. It employs more accurate approximations to find the best models and an advanced regularization technique, enhancing model training speed and generalization and reducing model complexity (20). Next, we sought to understand the associations between lymphopenia and baseline features, including baseline blood counts, clinical and tumor characteristics, treatment regimens and especially radiation dose, in patients with breast cancer who underwent radiation therapy. Therefore, we assessed the relative importance and direction of feature contribution to lymphopenia in XGboost models *via* Shapley additive explanation (SHAP) approaches (21, 22). Rigorous quality validations were implemented, including cross-validation, bootstrapping and Proof-of-concept clinical validation.

## Materials and Methods

### Description of Cohorts

Lymphopenia (grade ≥1) was defined from the Common Terminology Criteria for Adverse Events, version 4.0 (CTCAE v4.0). Patients with breast cancer who received adjuvant radiation therapy from March 2015 to October 2019 at the University of Hong Kong-Shenzhen Hospital formed the study population (Testing cohort). The eligibility criteria were: pathologically confirmed invasive breast cancer, received adjuvant radiation therapy, aged 18-years old or above, peripheral lymphocyte counts evaluated within 7 days after the end of radiation therapy in the same hospital. Exclusion criteria included patients with non-invasive breast cancer (stage 0), stage IV or recurrent breast cancer, breast lymphoma, and underlying autoimmune diseases. Patients who underwent surgery and chemotherapy in other hospitals were eligible if they received the whole course of radiotherapy at the University of Hong Kong-Shenzhen Hospital.

Another independent prospective validation cohort included patients between November 2019 and December 2020. Patients with breast cancer who underwent radiation therapy with the same criteria as the Testing cohort were selected from a prospective observational study of the Bio-Imaging Repository Databank (BIRD) project at the University of Hong Kong-Shenzhen Hospital.

### Radiation Therapy Procedures

Radiotherapy techniques included 2-field tangential opposing technique (2D-fields), tangential opposing fields with an anterior SCF field (three-dimensional conformal technique, 3D-fields) and RapidArc (Varian Medical Systems, Palo Alto, CA, USA). CT scans from the skull base to the level of the first lumbar vertebra were obtained in this study. The 2D-fields technique was normally employed on patients who needed irradiation only of the breast. 3D-fields technique was usually used on patients who needed irradiation of the breast or chest wall and supraclavicular fossa or axillary fossa. RapidArc, a volume modulated arc therapy technique, was employed on patients with invasive breast cancer with N3 or N2 diseases with centrally or medially located primary tumors.

### Feature Groups

The “clinical data” feature group included age, family disease (without, with), smoking (without, with) and drinking (without, with), and menopausal (pre, peri, post). All participants are females.

The “tumor characteristics” feature group included modified N stage (0, more than 0), modified stage (I, II, III), tumor sides (right, left), tumor size, estrogen receptors (ER) (negative, positive), progesterone receptors (PR) (negative, positive), human epidermal growth factor receptor 2 (HER2) (negative, positive), immunohistochemistry (IHC) (HR+/HER2-, HR-/HER2+, HR+/HER2+, HR-/HER2-) and Ki67.

The “blood cells” feature group included the baseline counts of white blood cell (WBC), hemoglobin (Hb), platelet (PLT), neutrophil (ANC), lymphocyte (LYM) and monocyte (MON), which were measured within 7 days before radiation therapy.

The “radiotherapy” feature group included the total body dose, mean heart dose, maximum heart dose, V20 of ipsilateral lung, V5 of ipsilateral lung, mean ipsilateral lung, V20 of the bilateral lung, V5 of the bilateral lung, mean bilateral lung, radiotherapy technique (RapidArc, 2D-fields, 3D-fields), electron boost (none, 10Gy/5fx, 16Gy/8fx), RT fields (tangential breast only, breast or chest wall with regional lymphatics) and RT dose (40.5Gy/15fx, more than 50Gy/25fx). Irradiation dose and volume were extracted from a dose-volume histogram (DVH).

The “treatment” feature group included treatment regimens (breast-conserving therapy (BCT), modified radical mastectomy (MRM)), surgical treatment (Sentinel lymph node biopsy (SLNB), axillary lymph node dissection (ALND)), margin (clear, close or positive), chemotherapy strategy (none, neoadjuvant, adjuvant, both neoadjuvant and adjuvant), chemotherapy regimens (taxane, anthracycline+taxane, others), anti-HER2 therapy (without, with), endocrine therapy (without, with).

### Predictive Models of the Lymphopenia Events

We established XGboost models by tenfold cross-validation (CV) framework *via* 100 bootstrapping iterations to predict radiation-induced lymphopenia. We used either all features or each feature group as input to build XGboost models and applied Lasso regressions for comparison. To estimate the explained prediction variances, we evaluated the predicting results using sensitivity, specificity, accuracy, f1-score, the area under the receiver operating characteristic curve (ROC-AUC) and the area under the precision-recall curve (PR-AUC). All samples were used in training and validation, considering XGboost’s abilities to handle the missing data. In contrast, those samples without missing data were used in training and validating the Lasso regressions.

In each bootstrapping iteration, we randomly selected 80% of patients and grouped them into one patient set. The patient set was separated into a training set and a testing set (ratio 7: 3) and performed the tenfold CV to train models. Next, AUC and its minimum value were used as hyperparameters for Lasso regression in the training set. The grid-search sets were used as hyperparameters for XGboost, i.e. learning_rate from 0.01 to 0.1 step 0.01, gamma from 0 to 5 step 1, max_depth from 3 to 6 step 1, scale_pos_weight from 1 to 2 step 0.2, subsample from 0.7 to 1 step 0.1, colsample_bytree from 0.7 to 1 step 0.1, min_child_weigth from 3 to 6 step 1, max_delta_step from 0 to 5 step 1, the other hyperparameters were set as defaults. Finally, we computed the model’s prediction on the remaining testing set.

### Feature Attributions Analysis

To emphasize the predictive power of XGboost models, we used Shapley additive explanation (SHAP). SHAP values assign an importance value to each feature representing the effect on the model prediction. In brief, for a specific prediction, the SHAP value of a feature is defined as the change in the expected value of the model’s output when this feature is observed versus when it is missing.

We computed individual SHAP values using the module TreeExplainer (Python v.0.37.0). We summarized the mean absolute SHAP value across all instances, reflecting the mean effect of each feature on predicting the lymphopenia outcome and serving as a feature’s contribution measure. The bigger mean absolute SHAP value mentions the feature is more important in lymphopenia.

We further determined the directional mean absolute SHAP values by taking into account the mean value of Spearman correlations between individual SHAP values and corresponding outcomes *via* all iterations. The directional SHAP values more closing to 1 or -1 mentions that the feature promotes or protects the occurrence of lymphopenia more. The directional SHAP values has the same sign meaning as the odd ratio in the Lasso regression. Finally, we used a graphical layout (Cytoscape 3.7.2) in order to visualize the contributions of features (both mean absolute SHAP value and conditional SHAP values) in XGboost models for the event of lymphopenia.

### Selection of Paired Patients

To study the contributions of the less important features that important features may overshadow, we selected the paired patients with controlled discrepancy of important features in the Testing cohort and Validation cohort separately. In this study, for every two patients a and b, the discrepancy was defined as the mean value of absolute relative differences in important features: $\sum_{i=1}^{n}abs\;(\frac{f_{a}-f_{b}}{f_{max}})/n$ , where i is the important feature number, n is the total important feature number, abs means absolution, f_a and f_b are the feature values in patients a and b, respectively, and f_max means the maximum of the feature. For each less important feature we need to study, the important features were defined as those with the SHAP values bigger than the less important features. Two patients with a discrepancy less than the threshold and different lymphopenia outcomes were considered one paired patient. In the selected paired patient cohort, we used paired t-test to assess the significance of the less important feature on the lymphopenia events.

### Statistical Analysis

For all statistical analysis and prediction models we used R 3.6.1 (23) with the following packages: ggplot2 3.3.2, glmnet 4.0.2, caret 6.0.86, xgboost 1.2.0.1, Matrix 1.2.18; For SHAP value analysis we used Python 3.7.3 (24) with the following packages: pandas 0.24.2, numpy 1.18.5, sklearn 0.20.3, shap 0.37.0, xgboost 1.3.1. In univariate analysis, P values were corrected by Bonferroni correction. Statistical significance was defined as P values <0.05.

## Results

### Patients and Characteristics

**Figure 1:**
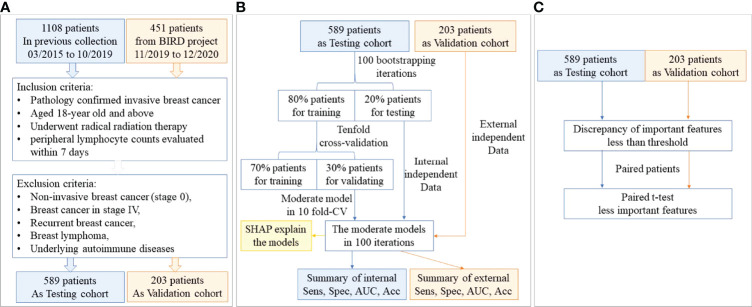
The study flowchart. **(A)** The patient flowchart; **(B)** the machine learning flowchart; **(C)** the statistical verification flowchart.

The patient flowchart is shown in **Figure 1A**. In the Testing cohort, 589 patients with breast cancer who underwent radiation therapy were enrolled. We collected data about clinical characteristics, baseline and post-treatment blood test results, tumor characteristics, radiation dose and other therapy regimes (summarized in **Table 1**). All patients were females with a median age of 45 (1st to 3rd Qu: 39-51). The median lymphocyte count in patients before radiation therapy was 1530/µL (1st to 3rd Qu: 1200 to 1860), while it decreased to 950/µL (1st to 3rd Qu: 750 to 1170) after radiation therapy. A total of 340 (57.7%) patients had lymphopenia (grade≥1) after radiation therapy.

**Table 1:** The characteristics of breast cancer patients in Testing cohort (total 589 patients), continues features are shown as median (1st to 3rd quantile) and classified features are shown as numbers (percentage).

Feature	Subgroup	Median (1st - 3rd Qu) or number (percentage)		Odd ratio (95%CI)	*P* value	Adjusted P value
		Without lymphopenia	With lymphopenia			
**lymphopenia**		249	340			
**baseline white blood cells**		5.76(4.82-6.95)	4.9(3.87-5.96)	0.85(0.78-0.92)	<0.001	<0.001
**baseline hemoglobin**		120(110-128)	117.5(110-124)	0.98(0.97-1)	0.024	1
**baseline platelet**		244(212-282)	227.5(185.8-271)	0.99(0.99-1)	0.003	0.14
**baseline neutrophil**		3.49(2.75-4.38)	2.98(2.22-3.98)	0.92(0.85-0.99)	0.036	1
**baseline lymphocyte**		1.73(1.45-2.12)	1.34(1.09-1.66)	0.14(0.09-0.21)	<0.001	<0.001
**baseline monocyte**		0.34(0.25-0.42)	0.28(0.23-0.36)	0.12(0.04-0.36)	<0.001	0.012
**RT technology**	RapidArc	2 (0.8)	98(28.82)	reference		
	2D-fields	133 (53.41)	78(22.94)	0.012(0.001-0.039)	<0.001	<0.001
	3D-fields	114(45.78)	164(48.24)	0.029(0.004-0.095)	<0.001	<0.001
**RT fields**	Tangential breast only	133(53.41)	80(23.53)	reference		
	Breast/chest wall with regional lymphatics	116(46.59)	260(76.47)	3.73(2.62-5.32)	<0.001	<0.001
**RT Dose**	40.5Gy/15fx	241(96.79)	290(85.29)	reference		
	more than 50Gy/25fx	8(3.21)	50(14.71)	5.19(2.55-12)	<0.001	0.001
**electron**	none	92(36.95)	196(57.65)	11reference		
	10Gy/5fx	137(55.02)	129(37.94)	0.44(0.31-0.62)	<0.001	<0.001
	16Gy/8fx	20(9.03)	15(4.41)	0.35(0.17-0.72)	0.004	0.21
**mean heart dose**		1.92(0.42-2.81)	2.25(0.53-4.31)	1.29(1.18-1.41)	<0.001	<0.001
**maximum heart dose**		40.68(3.8-41.76)	39.05(5.02-42.24)	1.01(1-1.02)	0.15	1
**integral dose of the total body**		3.56(2.99-4.06)	4.48(3.64-6.43)	2.27(1.91-2.73)	<0.001	<0.001
**V20 of ipsilateral lung**		17.4(12.5-23.5)	22.95(18.57-27.2)	1.1(1.08-1.13)	<0.001	<0.001
**V5 of ipsilateral lung**	** **	31.3(23.2-41.1)	43.25(34.05-60.73)	1.08(1.06-1.09)	<0.001	<0.001
**mean ipsilateral lung dose**		8.71(6.43-11.01)	11.59(8.94-13.22)	1.29(1.21-1.37)	<0.001	<0.001
**V20 of bilateral lungs**		8.6(6-11.4)	11.2(9-13.93)	1.2(1.15-1.27)	<0.001	<0.001
**V5 of bilateral lungs**		15.4(11.6-20)	22.3(16.68-39.12)	1.12(1.09-1.15)	<0.001	<0.001
**mean bilateral lungs dose**		4.34(3.26-5.4)	5.99(4.54-8.13)	1.57(1.43-1.74)	<0.001	<0.001
**age**		46(40-53)	44.5(38-51)	0.98(0.96-1)	0.028	1
**family history**	without	168(67.47)	217(63.82)	reference		
	with	81(32.53)	123(36.18)	1.18(0.83-1.66)	0.36	1
**smoking history**	without	190(76.31)	242(71.18)	reference		
	with	2(0.8)	1(0.29)	0.39(0.018-4.13)	0.45	1
	unknown	57(22.89)	97(28.53)	1.34(0.92-1.96)	0.13	1
**drinking history**	without	192(77.11)	240(70.59)	reference		
	with	0(0)	3(0.88)	1.69E6(2.25e-23-NA)	0.98	1
	unknown	57(22.89)	97(28.53)	1.36(0.94-1.99)	0.11	1
**menopausal**	premenopausal	156(62.65)	239(70.29)	reference		
	perimenopausal	18(7.23)	21(6.18)	0.76(0.39-1.49)	0.42	1
	postmenopausal	75(30.12)	80(28.53)	0.7(0.48-1.01)	0.057	1
**modified Nstage**	0	135(54.22)	89(26.18)	reference		
	more than 0	114(45.78)	251(73.82)	3.34(2.37-4.74)	<0.001	<0.001
**modified stage**	I	96(38.55)	55(16.18)	reference		
	II	117(46.99)	149(43.82)	2.22(1.48-3.36)	<0.001	0.007
	III	36(14.46)	136(40)	6.59(4.06-10.9)	<0.001	<0.001
**tumor sides**	tumor side at left	139(55.82)	159(46.76)	reference		
	tumor side at right	110(44.18)	181(53.23)	1.42(1.02-1.97)	0.038	1
**tumor size**		2(1.3-2.5)	2(1.38-2.8)	1.13(1-1.28)	0.047	1
**ER**	-	66(26.51)	97(28.53)	reference		
	+	183(73.49)	243(71.47)	0.9(0.62-1.3)	0.59	1
**PR**	-	85(34.14)	128(37.65)	reference		
	+	164(65.86)	212(62.35)	0.86(0.61-1.21)	0.38	1
**HER2**	-	176(70.68)	259(76.18)	reference		
	+	73(29.31)	81(23.82)	0.75(0.52-1.09)	0.13	1
**IHC**	HR+/HER2-	143(57.43)	203(59.71)	reference		
	HR-/HER2+	29(11.65)	35(10.29)	0.85(0.5-1.46)	0.55	1
	HR+/HER2+	43(17.27)	48(14.12)	0.79(0.49-1.25)	0.31	1
	HR-/HER2-	34(13.65)	54(15.88)	1.12(0.69-1.82)	0.65	1
**Ki67**		30(15-40)	30(15-40)	1(0.99-1.01)	0.98	1
**surgery regimens**	BCT	151(60.64)	135(39.71)	reference		
	MRM	98(39.36)	205(60.29)	2.34(1.68-3.28)	<0.001	<0.001
**surgery regimens**	SLNB	127(51)	77(22.65)	reference		
	ALND	122(49)	263(77.35)	3.56(2.5-5.09)	<0.001	<0.001
**margin**	clear margin	235(94.38)	326(95.88)	reference		
	close or positive margin	14(5.62)	14(4.12)	0.72(0.34-1.55)	0.39	1
**chemotherapy strategy**	none	41(16.48)	17(5)	reference		
	neoadjuvant	26(10.44)	78(22.94)	7.24(3.59-15.2)	<0.001	<0.001
	adjuvant	177(71.08)	236(64.41)	3.22(1.8-5.99)	<0.001	0.007
	neoadjuvant+adjuvant	5(2)	9(2.65)	4.34(1.31-16)	0.019	0.98
**chemotherapy regimens**	others	45(18.07)	28(8.24)	reference		
	taxane	82(32.93)	61(17.94)	1.2(0.67-2.14)	0.54	1
	anthracycline+taxane	122(49)	251(73.82)	3.31(1.98-5.61)	<0.001	<0.001
**anti Her2 therapy**	without	182(73.09)	260(76.47)	reference		
	with	67(26.91)	80(23.53)	0.84(0.57-1.22)	0.35	1
**endocrine therapy**	without	63(25.3)	89(26.18)	reference		
	with	186(74.7)	251(73.82)	0.96(0.66-1.39)	0.81	1

Odd ratio (95% confident interval) and corresponding P values in logistical regression for the events of lymphopenia, the adjusted P values are P values after Bonferroni correction. RT, radiation treatment; ER, estrogen receptors; PR, progesterone receptors; IHC, immunohistochemistry; HR, hormone receptor; HER2, human epidermal growth factor receptor 2; BCT, breast-conserving therapy; MRM, modified radical mastectomy; SLNB, Sentinel lymph node biopsy; ALND, axillary lymph node dissection.

To validate the accuracy and robustness of our results, we adopted an independent prospective cohort (Validation cohort) enrolling 203 patients with breast cancer. The patients in the Validation cohort were also all females with a median age of 45 (1st to 3rd Qu: 39-51). The median lymphocyte count in patients before radiation therapy was 1500/µL (1st to 3rd Qu: 1230 to 1935), while it decreased to 1000/µL (1st to 3rd Qu: 700 to 1255) after radiation therapy. A total of 104 (51.2%) patients had lymphopenia (grade≥1) after radiation therapy. The characteristics of the Validation cohort were all summarized in **Table S1**.

### Prediction of the Event of Lymphopenia

The flowchart for establishing the machine learning models is shown in **Figure 1B**. We trained XGboost and lasso regression in tenfold CV *via* 100 bootstrapping iterations in the Testing cohort to predict the binary classified lymphopenia. The models were validated in the Validation cohort across all iterations. The final XGboost models were development with the hyperparameters as follows: learning_rate=0.04, gamma=5, max_depth=3, scale_pos_weight=1.8, subsample=0.8, colsample_bytree=0.7, min_child_weigth =5, max_delta_step = 1.

**Figure 2:**
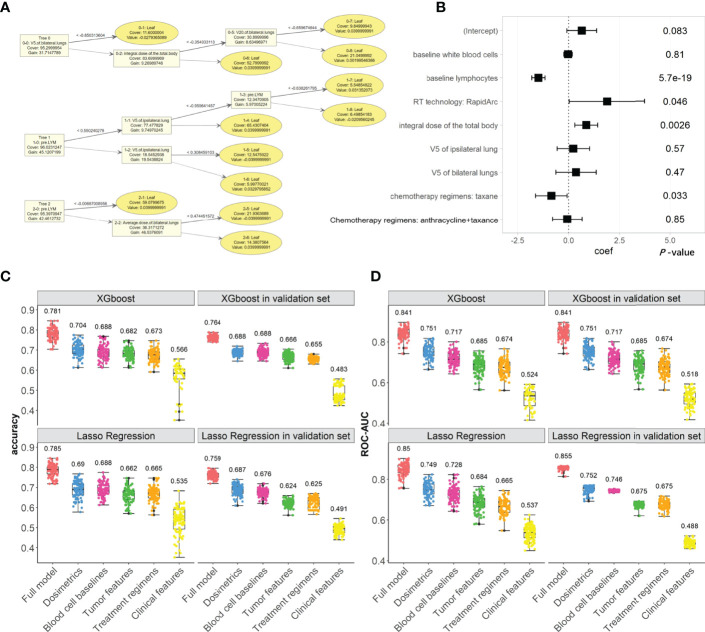
The XGboost and Lasso regression models for predicting the radiation-induced lymphopenia were trained in Testing cohort and validated in Validation cohort. **(A)** The top two trees in one example of XGboost model; **(B)** coefficients and *P*-values in one example of Lasso regression; **(C, D)** the classifying performances (accuracy and ROC-AUC, respectively) of XGboost models across all iterations in Testing cohort and in Validation cohort, compared with the Lasso regressions. In subplots **(C, D)**, numerical labels are median values. The color represents the feature’s group, including: the full model (Orange), dosimetrics (blue), blood cell baselines (maroon), tumor features (green), Treatment regimens (Khaki), clinical features (yellow).

In the full XGboost models, the main metrics to evaluate the classifying abilities are both accuracy (median: 0.781, 1st to 3rd Qu: 0.762 to 0.817) and ROC-AUC (median: 0.841, 1st to 3rd Qu: 0.822 to 0.856); the models were validated in Validation cohort for accuracy (median: 0.764, 1st to 3rd Qu: 0.753 to 0.774) and ROC-AUC (median: 0.841, 1st to 3rd Qu: 0.817 to 0.868), as shown in **Figure 2**. Other evaluation metrics in the Testing and Validation cohorts are shown in **Figures S1–S4** and compared, sensitivity, specificity, F1-score, and PR-AUC. The XGboost models’ evaluation metrics and prediction abilities in the Validation cohort keep up with the Lasso regression’s metrics.

We also investigated the abilities of each feature group to predict lymphopenia. Radiation dose and baseline blood cells were the two important feature groups, as shown in **Figure 2**. In contrast, the other feature groups, including tumor characteristics, treatment regimens and clinical features, have fewer contributions to lymphopenia. These are similar to the results of lasso regressions, which are also shown in **Figure 2** and **Figures S1–S4**.

### Feature Attributions

The feature parameters, including both gain and frequency indexes, in the full XGboost models constructed *via* all iterations, are summarized in **Table S2**. In each XGboost model, the gain index represents the fractional contribution of each feature to the model based on the total gain of this feature’s splits; and the frequency index represents the relative number of times a feature has been used in sub-trees in one XGboost model. The higher gain and frequency indexes mean the more important predictive feature for predicting outcome. We also checked the features’ coefficients and P-values in Lasso regressions and their occurrence frequencies (**Table S3**). The top 10 important features for lymphopenia either in XGboost models or Lasso regressions are compared in **Figure S5**.

**Figure 3:**
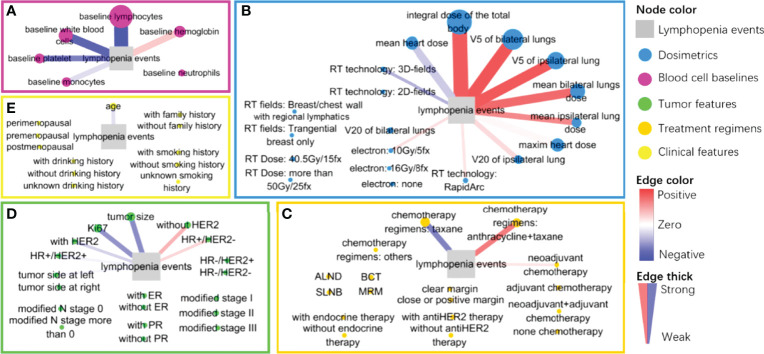
The graphic layout of directional SHAP values of each feature classified in five feature groups: **(A)** baseline blood cells group (maroon), **(B)** radiation dose group (blue), **(C)** treatment regimens group (Khaki), **(D)** tumor characteristics group (green) and **(E)** clinical characteristics group (yellow). The event of lymphopenia is a gray square. Each feature node is colored by group and sized by the SHAP values; each edge is colored from red (positive) to blue (negative) by the direction value of SHAP and its thickness is sized by the absolute direction values of SHAP.

However, both Gain and frequency indexes in XGboost cannot show the directional contributions of each feature on the occurrence of lymphopenia, which is not similar to the meaning of coefficient in Lasso regressions. We analyzed the directional SHAP values of each feature in the XGboost models, illustrated in the method section. The directional SHAP values of features in XGboost models are visualized in **Figure 3**. The comparison of feature contributions to the occurrence of lymphopenia between the directional SHAP values in XGboost models and the coefficients in Lasso regressions is listed in **Table 2**.

**Table 2:** The dummy features’ contributions to lymphopenia in Testing cohort (total 589 patients), including the mean SHAP value and direction of SHAP in XGboost models and the mean coefficients in Lasso regression across all iterations.

Feature	XGboost models		Lasso regressions	
	SHAP value	direction of SHAP	Coefficients	*P* value
baseline lymphocytes	5.226	-0.964	-0.902	<0.001
integral dose of the total body	4.021	0.858	0.487	<0.001
V5 of bilateral lungs	1.451	0.886	0.282	<0.001
V5 of ipsilateral lung	1.151	0.871	0.349	<0.001
baseline white blood cells	0.990	-0.838	-0.069	0.106
mean bilateral lungs dose	0.905	0.788	0.171	0.076
maxim heart dose	0.783	0.187	0	1
mean heart dose	0.774	-0.377	0	1
baseline hemoglobin	0.737	0.378	0.124	<0.001
baseline platelet	0.579	-0.681	-0.104	<0.001
baseline monocytes	0.533	-0.295	-0.063	0.048
mean ipsilateral lung dose	0.469	0.654	0.153	0.138
chemotherapy regimens: taxane	0.420	-0.672	-0.334	<0.001
V20 of ipsilateral lung	0.383	0.308	0	1
chemotherapy regimens: anthracycline+taxane	0.368	0.714	0.238	<0.001
tumor size	0.359	-0.629	0	1
Ki67	0.298	-0.600	-0.092	0.014
baseline neutrophils	0.283	0.053	0.061	1
age	0.254	-0.282	0.016	1
without HER2	0.160	0.470	0.290	<0.001
V20 of bilateral lungs	0.150	0.126	0.118	1
RT technology: 3D-fields	0.083	-0.477	0	1
RT technology: RapidArc	0.081	0.258	0.759	<0.001
with HER2	0.053	-0.341	0	1
neoadjuvant chemotherapy	0.050	0.256	0.321	<0.001
HR+/HER2-	0.045	0.321	0.159	1
electron: 10Gy/5fx	0.041	0.290	0.276	1
HR+/HER2+	0.040	-0.206	-0.245	0.016
modified stage II	0.040	0.054	-0.056	1
without drinking history	0.025	-0.142	-0.796	1
tumor side at left	0.024	-0.187	-0.103	0.306
without antiHER2 therapy	0.021	0.141	0	1
with family history	0.021	0.016	0.047	1
modified stage III	0.021	0.131	0.019	1
modified stage I	0.021	-0.094	0	1
without endocrine therapy	0.019	-0.082	-0.288	1
without PR	0.018	0.052	-0.195	1
adjuvant chemotherapy	0.016	-0.116	-0.105	1
premenopausal	0.015	0.109	0.187	1
without ER	0.015	-0.016	0.097	1
without smoking history	0.014	0.068	0	1
with antiHER2 therapy	0.012	-0.119	0	1
unknown drinking history	0.011	0.024	0	1
SLNB	0.011	0.001	0.150	1
RT technology: 2D-fields	0.010	-0.068	0	1
BCT	0.010	0.009	-0.300	1
unknown smoking history	0.009	0.048	0	1
with PR	0.008	-0.078	0	1
postmenopausal	0.008	-0.047	-0.151	1
ALND	0.008	0.034	0	1
with endocrine therapy	0.007	0.032	0	1
none chemotherapy	0.007	-0.043	-0.311	0.771
electron: none	0.006	0.000	0	1
HR-/HER2+	0.005	-0.046	-0.244	1
with ER	0.005	-0.040	0	1
without family history	0.004	-0.025	0	1
HR-/HER2-	0.004	0.026	0	1
chemotherapy regimens: others	0.003	0.001	0	1
tumor side at right	0.003	0.027	0	1
MRM	0.002	-0.018	0	1
modified N stage 0	0.001	0.000	0	1
electron: 16Gy/8fx	0.001	-0.004	-0.273	<0.001
RT fields: Breast/chest wall with regional lymphatics	0.001	0.017	0	1
neoadjuvant+adjuvant chemotherapy	0	0	-0.225	1
with drinking history	0	0	0	1
clear margin	0	0	0.391	<0.001
close or positive margin	0	0	0	1
perimenopausal	0	0	-0.281	0.76
modified N stage more than 0	0	0	0	1
RT Dose: 40.5Gy/15fx	0	0	-0.456	1
RT Dose: more than 50Gy/25fx	0	0	0	1
RT fields: Tangential breast only	0	0	0	1
with smoking history	0	0	-0.600	0.017

SHAP value is more than zero, the higher SHAP value the more contribution of feature to lymphopenia; direction of SHAP is range from -1 to 1, more promotive to lymphopenia when close to 1 while more protective when closet to -1. RT, radiation treatment; ER, estrogen receptors; PR, progesterone receptors; IHC, immunohistochemistry; HR, hormone receptor; HER2, human epidermal growth factor receptor 2; BCT, breast-conserving therapy; MRM, modified radical mastectomy; SLNB, Sentinel lymph node biopsy; ALND, axillary lymph node dissection.

### Baseline Blood Cells’ Attributions

As shown in **Figure 3A**, we considered the baseline blood cell counts’ contributions to the occurrence of lymphopenia in the XGboost and SHAP analysis. The baseline lymphocyte counts are negatively associated with lymphopenia events (SHAP = 5.226, direction of SHAP = -0.964). The higher the baseline lymphocyte level, the fewer lymphopenia events after treatment. Interestingly, baseline hemoglobin level promotes lymphopenia events (SHAP = 0.737, direction of SHAP = 0.378), while white blood cells, platelets and monocytes protected the patients from lymphopenia. We also found their directional contributions to the lymphopenia events were consistent with coefficients in Lasso regressions (**Table 2**)

### Dose Attributions

We considered the contribution of the radiation dose to the occurrence of lymphopenia in XGboost models and SHAP analysis, as shown in **Figure 3B**. The total body dose was an important radiation dose leading to the lymphopenia event (SHAP = 4.021, direction of SHAP = 0.858). Followed by V5 of bilateral lungs (SHAP = 1.451, direction of SHAP = 0.886), V5 of the ipsilateral lung (SHAP = 1.151, direction of SHAP = 0.871), mean dose of bilateral lungs (SHAP = 0.905, direction of SHAP = 0.788), maximum heart dose (SHAP = 0.783, direction of SHAP = 0.187), mean heart dose (SHAP = 0.774, direction of SHAP = -0.377), mean dose of the ipsilateral lung (SHAP = 0.469, direction of SHAP = 0.654), V20 of the ipsilateral lung (SHAP = 0.383, direction of SHAP = 0.308), and V20 of bilateral lungs (SHAP = 0.15, direction of SHAP = 0.126), The order of SHAP value almost agrees with the feature attribution results (**Figure S5** and **Table S2**).

**Figure 4:**
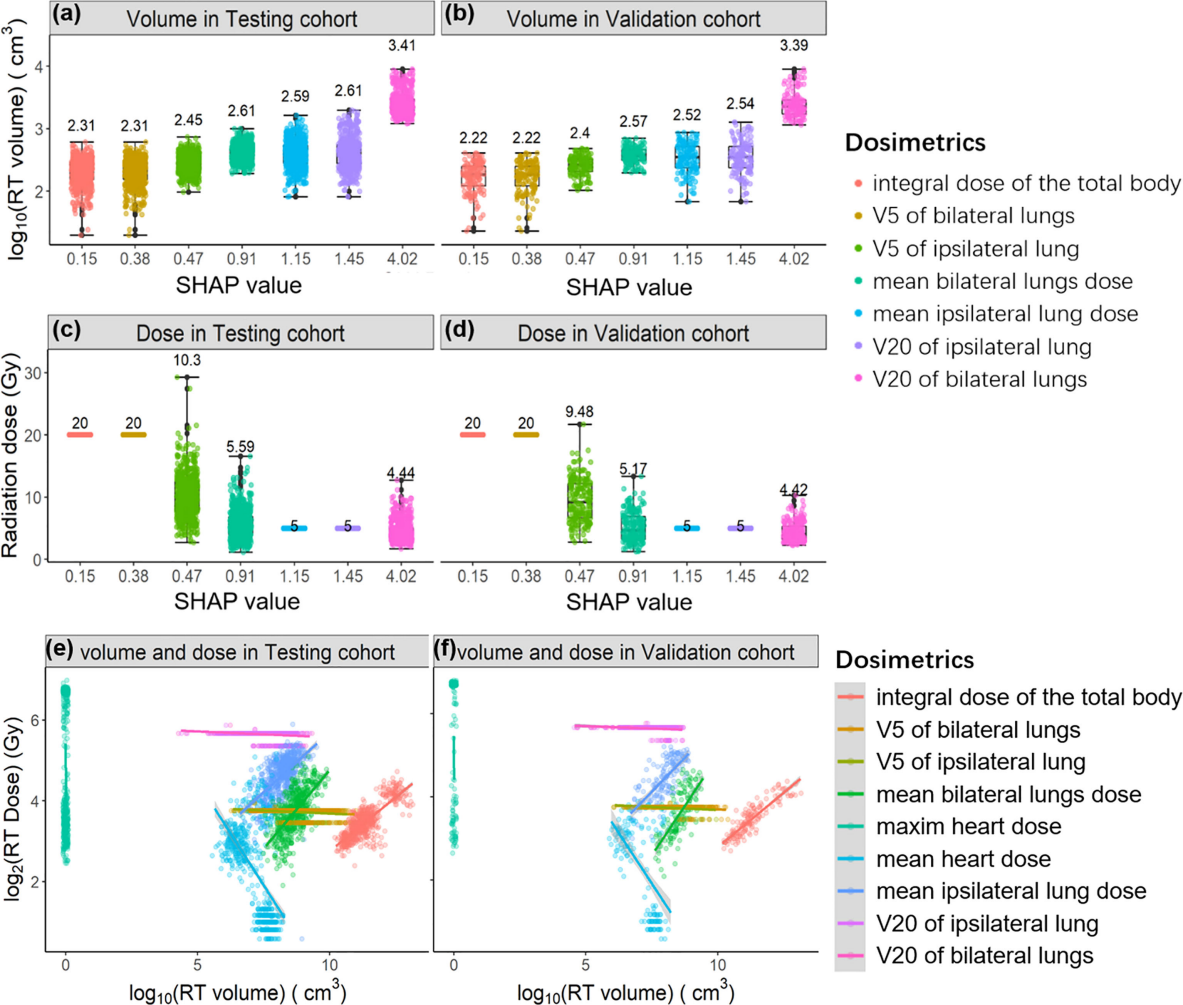
The relationships between SHAP value and irradiation volume in the Testing cohort **(A)** and in the Validation cohort **(B)**; the relationships between SHAP value and irradiation dose in the Testing cohort **(C)** and in the Validation cohort **(D)**. The regression relationships between irradiation volume and irradiation dose in the Testing cohort **(E)** and in the Validation cohort **(F)**. Each radiation dose is differently colored and sorted by SHAP value.

Next, we extracted both irradiation doses and corresponding volumes of each radiation dose from the patient’s DVH curves. As shown in **Figures 4A–D**, we sorted both the irradiation volume and dose by their contributions to lymphopenia (SHAP values). The irradiation volumes are ordered ascendingly by SHAP values (log10(volume)~1.325*SHAP value, P value<1e-8). In contrast, the corresponding irradiation dose is in the descending order with SHAP values (log2(dose)~0.603*SHAP value, P value<1e-8). The regression information is shown in **Figure S6**. It can be illustrated that (point 1) lymphocytes are sensitive to an irradiation dose lower than 4Gy, because the integral dose of body (median: 4Gy, 1st-3rd Qu: 3.3-5Gy) is found to be the most important dose in lymphopenia; (point 2) the irradiation volume plays a significantly greater role than the irradiation dose, in promoting lymphopenia.

We studied the relationship between irradiation dose and irradiation volume in both the Test and Validation cohort (**Figures 4E, F**). Irradiation volume is commonly positively correlated with irradiation dose. Notably, the irradiation volume is low when the mean heart dose is higher both in the Testing cohort and the Validation cohort that means the higher mean heart dose is related to the smaller irradiation range. According to the points 1 and 2 we summarized above, the protective role of mean heart dose against a lymphopenia event (SHAP = 0.774, direction of SHAP = -0.377) can be explained. That is, not the higher mean heart dose, but the smaller corresponding irradiation volume protects against a lymphopenia event.

In addition, the irradiation volume of the maximum heart dose is almost close to zero, and the positive SHAP value of the maximum heart dose (SHAP = 0.783, direction of SHAP = 0.187) mentions that the irradiation dose promotes the event of lymphopenia when the irradiation volume is fixed. Following the points above, it can be further illustrated that: (point 3) the irradiation dose promotes the occurrence of lymphopenia when the irradiation volume is controlled. Because of the negative correlations between irradiation volume and irradiation doe of heart, the associations between the mean/maximum heart dose and SHAP value do not follow the regression relationships, it can be compared between **Figures S6, S7**.

### Other Features’ Attributions

Finally, we considered the contributions of other features to the occurrence of lymphopenia (**Figures 3C–E**), especially those with relatively high SHAP value and direction of SHAP. Notably, the chemotherapy regimens (anthracycline/taxane) were found to promote the occurrence of lymphopenia (SHAP = 0.368, direction of SHAP = 0.714). Conversely, the Taxane monotherapy chemotherapy regimens were less of risk factor (SHAP = 0.420, direction of SHAP = -0.672). These findings were consistent with the results in Lasso regressions, as shown in **Table 2**.

### Proof-of-Concept Clinical Validation

We found that maximum heart dose and V20 of ipsilateral lung promoted the lymphopenia events (SHAP = 0.783, direction of SHAP = 0.187; SHAP = 0.383, direction of SHAP = 0.308, respectively). The mean heart dose protected the patients from lymphopenia (SHAP = 0.774, direction of SHAP = -0.377) (**Table 2**). However, these three radiation doses were never included in Lasso regressions. The mean heart dose promotes lymphopenia in the univariate logistic analysis in both the Testing cohort (**Table 1**) and the Validation cohort (**Table S1**).

The contradiction between mean heart dose in SHAP analysis and univariate logistic analysis might be caused by the multi-collinearity among radiation doses (Pearson’s correlations > 0.181, as shown in **Figure S8**). Moreover, the contradiction between these three radiation doses in SHAP analysis and the lasso regressions may be because of the shrinkage function in lasso regression, which means the highly correlated but less important features may not be selected in the regression model. Therefore, we did a proof-of-concept analysis in paired patients for these three radiation doses, as illustrated in the method section and shown in **Figure 1C**.

**Figure 5:**
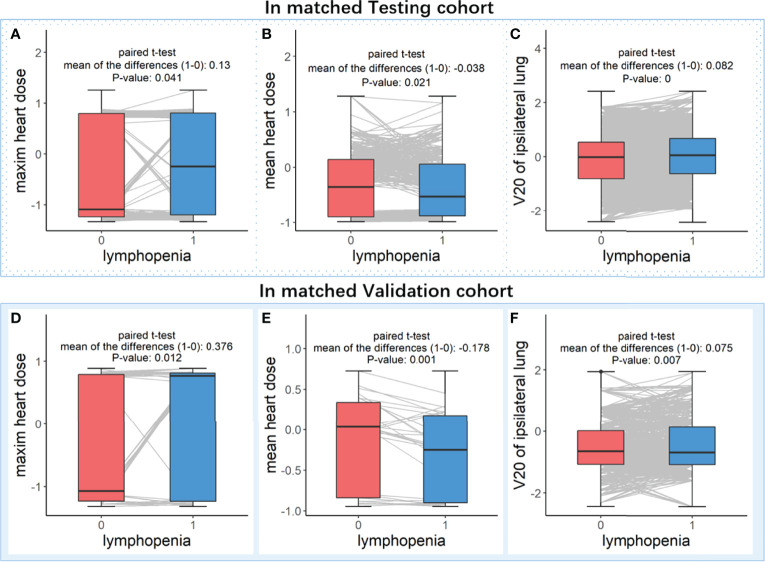
Paired t-test analysis in matched patients who with controlled discrepancy of important features. Subplots **(A–C)** are analyzed in the matched patients in Testing cohort, and subplots **(D–F)** are analyzed in the matched patients in Validation cohort. In the paired patients of each cohort, three boxplots (from left to right) were the comparisons of feature levels between with (blue box) and without (red box) lymphopenia in (**A** or **D**) maxim heart dose, (**B** or **E**) mean heart dose and (**C** or **F**) V20 of ipsilateral lung, respectively.

As shown in **Figure 5**, after controlling for the main features, the mean heart dose is higher in the patients without lymphopenia events (both P values < 0.05). In comparison, the maximum heart dose and V20 of the ipsilateral lung are significantly higher in the patients with lymphopenia events (all P values < 0.05). These results are all consistent with the SHAP value analysis, indicating that our XGboost models and directional mean absolute SHAP values rigorously revealed the features’ importance and their directional contribution to lymphopenia, especially those features were contradictory in univariate analysis or those features were never included in lasso regressions because of being masked by the highly correlated major features.

## Discussion

To best of our knowledge, our study is the first to describe the diverse potential determinants, especially including the complex radiation dose, of radiation-induced lymphopenia in the patients with breast cancer by the machine learning algorithm (XGboost). Furthermore, XGboost and SHAP interpretation approach were combined to determine the predictive performances and the feature contributions in radiation-induced lymphopenia. We found that baseline lymphocyte counts protect against while the baseline hemoglobin level impact the event of radiation-induced lymphopenia; more importantly, we summarize some regularities between radiation dose and occurrence of lymphopenia, i.e. (1) lymphocytes are sensitive to an irradiation dose lower than 4Gy; (2) the irradiation volume plays a significantly greater role than the irradiation dose, in promoting lymphopenia; (3) the irradiation dose promotes the occurrence of lymphopenia when the irradiation volume is controlled.

The protective role of baseline lymphocyte counts in radiation-induce lymphopenia is consistent to the common acknowledgement. However, there were little previous works have correlated the hemoglobin and lymphopenia in radiation therapy. It is known for decades that cancer-related hypoxia and anemia are associated with decrease in radiosensitivity of tumor cells (25). In another words, it can be hypothesized that hypoxia and anemia might decrease the radiosensitivity of lymphocytes which deserves more studies in the future. For the protective effects of platelets and monocytes, frequent platelet donation is usually associated with T-cell lymphopenia in platelet donation studies (26, 27), which mentioned that possible casual correlations between platelet reduction and lymphopenia but the mechanisms were still unknown. The correlations between monocytes and lymphopenia were seldom studied, but macrophage, which differentiated from monocyte, leads to either radiosensitization or radioresistance depending on different tumor types or different radiation regimen studied, and various molecular players as NF-kB, MAPKs, p53, reactive oxygen species, and inflammasomes that have been involved in these processes (28), which might mention the monocyte reduction correlated to lymphopenia at some extend.

Considering the impact of irradiation dose on lymphopenia, the contribution of integral dose of the total body also has been presented in our previous work of EDIC formula containing integral dose of the total body resulted lymphopenia in 488 esophageal cancer patients (29); V5 of ipsilateral lung/bilateral lungs have been previously reported to impact lymphopenia in non-small cell lung cancer patients (30). Similar findings have been reported in patients with early-stage lung cancer, V10 to V50 were significantly negatively correlated with the decrease of absolute lymphocyte counts following radiation (31). In patients with pancreatic cancer, V10, V15, and V20 were significantly higher in patients with severe lymphopenia (32), and the same findings in patients with esophageal squamous cell carcinoma (33). For the contribution of irradiation volume at heart, our findings are consistent with our previous study in lung cancer patients, i.e. higher heart V5 (the heart volume in 5Gy) was significant with decrease in post-radiation lymphocyte counts (31). The results of these studies were all consistent with our findings.

We illustrated that the irradiation volume had a more significant lymphotoxic impact than the irradiation dose. The lymphocytes behave as mobile organs and circulate through the blood at about 1 cycle/min (34). Therefore, this may explain our novel findings that the higher the volume of radiotherapy dose delivered may result in a higher administration of radiotherapy to the circulating lymphocytes, thereby increasing the risk of radiation-induced lymphopenia.

The impact of chemotherapy on lymphopenia is known. Our findings is consistent with findings from study of Tolaney SM et al. who demonstrated that lymphopenia (grade 3 or grade 4) was associated with the combined use of adjuvant anthracycline and taxane regimens in three breast cancer patient cohorts (35). This study revealed that the taxane only chemotherapy seemed to have less risk on lymphopenia (OR 1.2, 95%CI 0.67-2.14, P-value>0.5, as shown in **Table 1**) in univariate analysis which was consistent with our previous study (36), similarly in multivariable analysis. Less lymphopenia effect from taxane monotherapy may be simply explained by the less damage from the combination of anthracycline and taxane. Of note, we previously noted that single-agent taxane treatment increased serum IL-2 levels in patients with advanced breast cancer (37). IL-2 serves as a cell-cycle progression signal for T lymphocytes, stimulating their proliferation and differentiation. The underpinning biology driving chemotherapy-induced lymphopenia is not fully understood, and this study further highlights the unmet need for future studies.

There are some limitations in this study. First, we used an independent prospective cohort with different population and study protocols but the same staffs who assembled these two cohorts. Our findings are needed to be verified with external cohorts in the future. Secondly, it is well known that inflammation indicators are also important in immune responses including lymphopenia, while the Testing cohort in this study were established without inflammation indictors. This limitation could be addressed in subsequent studies by measuring inflammation indicator levels in serum.

In conclusion, in patients with breast cancer who underwent radiotherapy, we found that the baseline lymphocyte, platelet and monocyte play protective roles in lymphopenia; the usage of taxane results in less impact on lymphopenia than the combination of an anthracycline with a taxane; all radiation doses promote the occurrence of lymphopenia except the mean heart dose. Especially for the contributions of complicated radiation dose on lymphopenia, we draw three conclusions: 1) lymphocytes are sensitive to an irradiation dose lower than 4Gy; 2) the irradiation volume plays a more important role in promoting the occurrence of lymphopenia than the irradiation dose; 3) the irradiation dose promotes the lymphopenia occurrence when the irradiation volume is controlled. Higher than the dose’s priority, irradiation volume should be kept as small as possible during the planning process to avoid radiation-induced lymphopenia as long as the target coverage is not compromised.

## Data Availability Statement

The original contributions presented in the study are included in the article/**Supplementary Material**. Further inquiries can be directed to the corresponding author.

## Ethics Statement

The studies involving human participants were reviewed and approved by the ethics committee of the University of Hong Kong-Shenzhen Hospital. The patients/participants provided their written informed consent to participate in this study.

## Author Contributions

Conceptualization: HY, FC and F-MK; Data selection and clinical trials: FC and LY; Methodology: HY and YW; Funding acquisition: HY and F-MK; Project administration: K-OL and F-MK; Supervision: J-YJ; Writing – original draft: HY and FC; Writing – review and editing: K-OL, AH, and F-MK. All authors contributed to the article and approved the submitted version.

## Funding

This project was supported in parts by Shenzhen Fundamental Research Program (No. JCYJ2020109150427184), Shenzhen Science and Technology Program (No. KQTD20180411185028798).

## Conflict of Interest

The authors declare that the research was conducted in the absence of any commercial or financial relationships that could be construed as a potential conflict of interest.

## Publisher’s Note

All claims expressed in this article are solely those of the authors and do not necessarily represent those of their affiliated organizations, or those of the publisher, the editors and the reviewers. Any product that may be evaluated in this article, or claim that may be made by its manufacturer, is not guaranteed or endorsed by the publisher.
