# Supplementary material for: Potential Determinants for Radiation-Induced Lymphopenia in Patients With Breast Cancer Using Interpretable Machine Learning Approach
Source: Front Immunol. 2022 Jun 21;13:768811. doi: 10.3389/fimmu.2022.768811 (PMC9253393; doi:10.3389/fimmu.2022.768811)
Supplement: Supplementary file 1 [file DataSheet_1.zip › final files/Fig S1.docx]

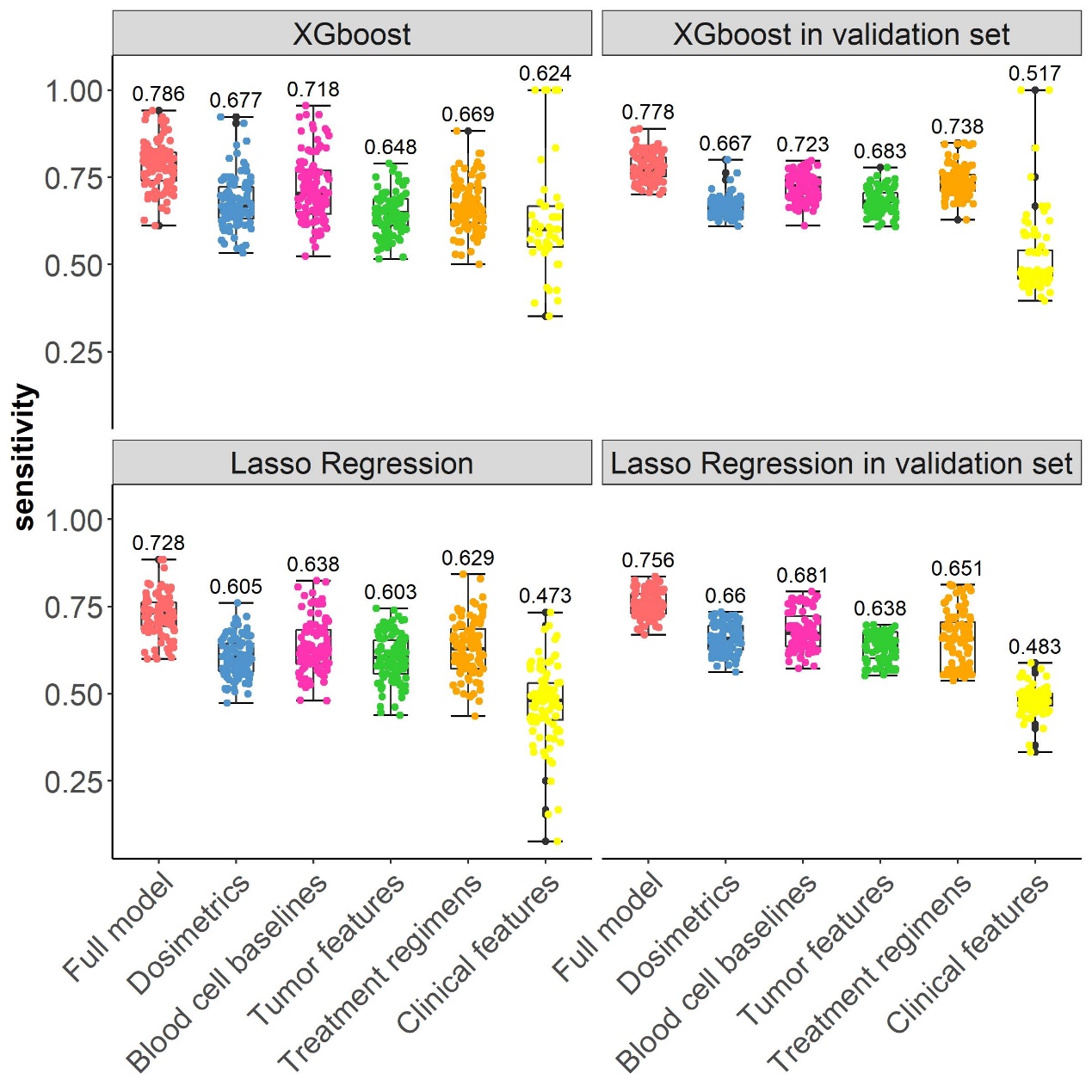
Fig S1. The sensitivity performances of full model and five feature groups in XGboost across all iterations. The XGboost models trained in Testing cohort for predicting the event of lymphopenia and validated in Validation cohort. The performance index of sensitivity is better when close to 1; In all subplots, numerical labels are median values. The color represents the feature’s group, including: the full model (Orange), dosimetrics (blue), blood cell baselines (maroon), tumor features (green), Treatment regimens (Khaki), clinical features (yellow).
