# Supplementary material for: Potential Determinants for Radiation-Induced Lymphopenia in Patients With Breast Cancer Using Interpretable Machine Learning Approach
Source: Front Immunol. 2022 Jun 21;13:768811. doi: 10.3389/fimmu.2022.768811 (PMC9253393; doi:10.3389/fimmu.2022.768811)
Supplement: Supplementary file 1 [file DataSheet_1.zip › final files/Fig S5.docx]

**
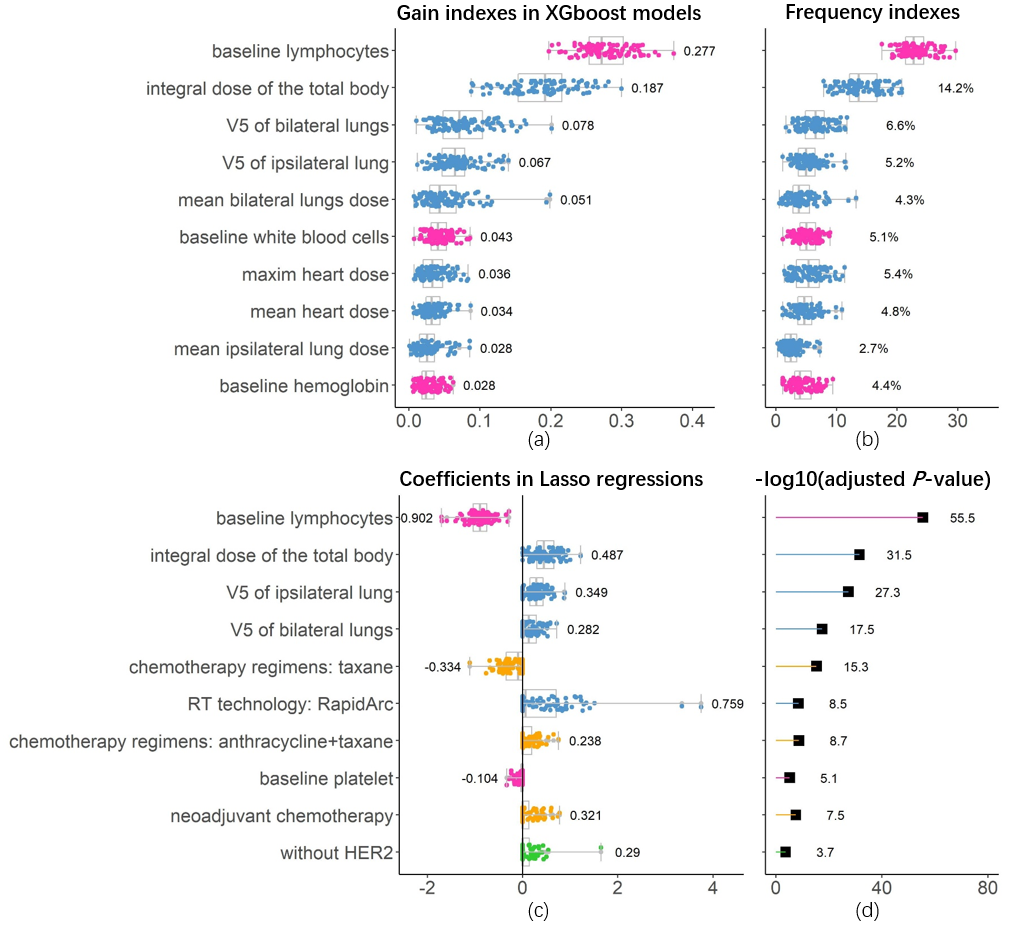
**

Fig S5. In full model, (a) the summarized gain indexes and (b) the frequency indexes via all iterations in XGboost models; (c) the summarized coefficients and (d) the corresponding *P*-value (after Bonferroni correction) in Lasso regressions. Both in the full XGboost models and Lasso regressions, we selected the top 10 of most appeared features to be shown. All detailed information was listed in Table S2 and 3. The color represents the feature’s group, including: the full model (Orange), dosimetrics (blue), blood cell baselines (maroon), tumor features (green), Treatment regimens (Khaki), clinical features (yellow).
