# Supplementary material for: Potential Determinants for Radiation-Induced Lymphopenia in Patients With Breast Cancer Using Interpretable Machine Learning Approach
Source: Front Immunol. 2022 Jun 21;13:768811. doi: 10.3389/fimmu.2022.768811 (PMC9253393; doi:10.3389/fimmu.2022.768811)
Supplement: Supplementary file 1 [file DataSheet_1.zip › final files/Fig S6.docx]

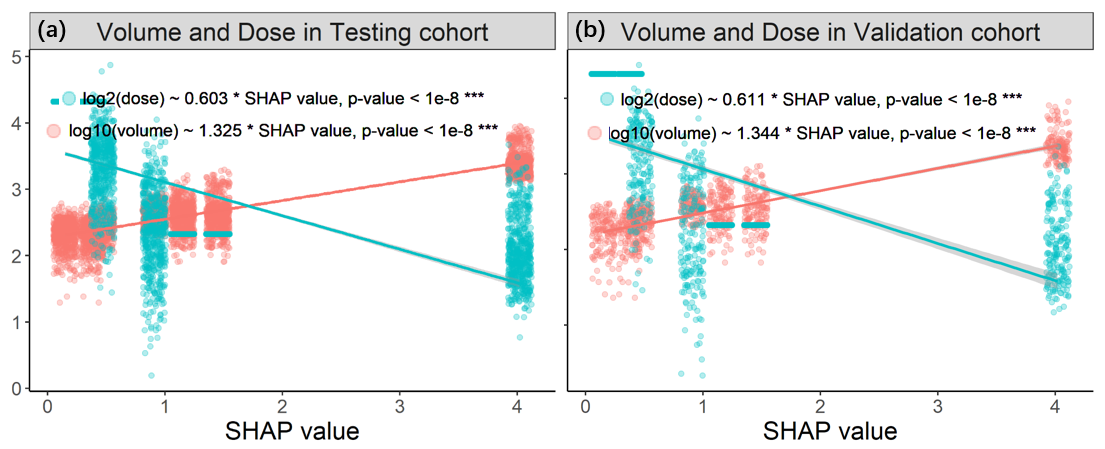
Fig S6. The regression relationships between SHAP and dose (Gy) transformed in log2, between SHAP and volume (cm^3^) transformed in log 10, in the Testing cohort (a) volume and in the Validation cohort (b)
