# Supplementary material for: Potential Determinants for Radiation-Induced Lymphopenia in Patients With Breast Cancer Using Interpretable Machine Learning Approach
Source: Front Immunol. 2022 Jun 21;13:768811. doi: 10.3389/fimmu.2022.768811 (PMC9253393; doi:10.3389/fimmu.2022.768811)
Supplement: Supplementary file 1 [file DataSheet_1.zip › final files/Fig S7.docx]

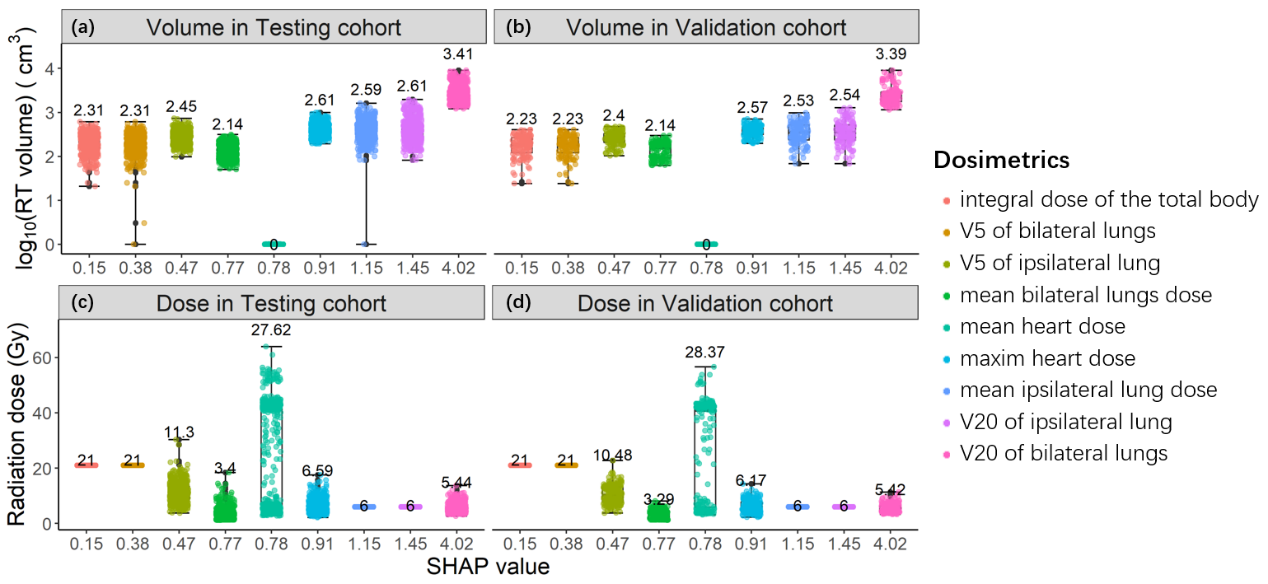
Fig S7. The boxplots for relationships between SHAP value and irradiation volume in the Testing cohort (a) and in the Validation cohort (b); the relationships between SHAP value and irradiation dose in the Testing cohort (c) and in the Validation cohort (d). It includes all dosimetrics especially maxim heart dose and mean heart dose.
