# Supplementary material for: Potential Determinants for Radiation-Induced Lymphopenia in Patients With Breast Cancer Using Interpretable Machine Learning Approach
Source: Front Immunol. 2022 Jun 21;13:768811. doi: 10.3389/fimmu.2022.768811 (PMC9253393; doi:10.3389/fimmu.2022.768811)
Supplement: Supplementary file 1 [file DataSheet_1.zip › final files/Fig S8.docx]

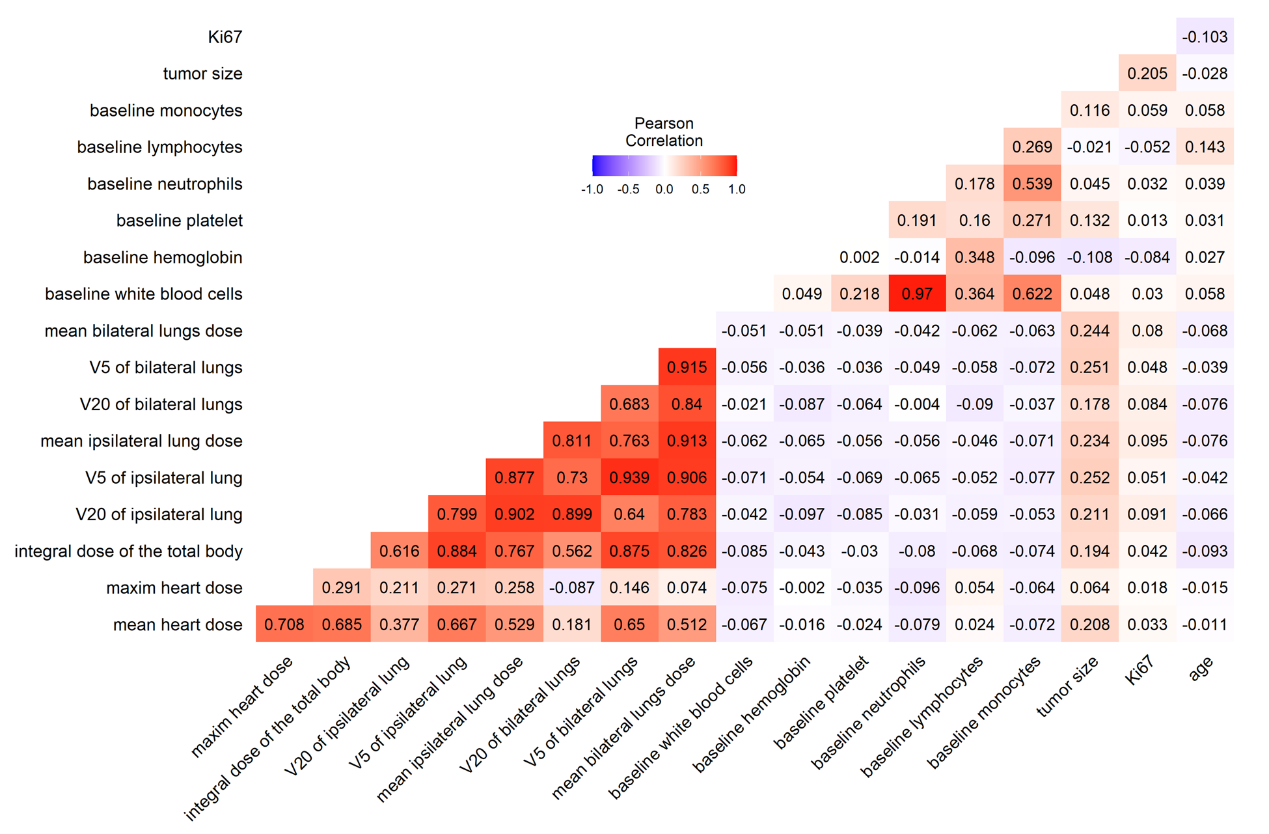
Fig S8. The Pearson’s correlations among all continues features. More positive correlation when close to 1 (red); more negative correlation when close to -1 (blue).
