# Supplementary material for: Potential Determinants for Radiation-Induced Lymphopenia in Patients With Breast Cancer Using Interpretable Machine Learning Approach
Source: Front Immunol. 2022 Jun 21;13:768811. doi: 10.3389/fimmu.2022.768811 (PMC9253393; doi:10.3389/fimmu.2022.768811)
Supplement: Supplementary file 1 [file DataSheet_1.zip › final files/Table S1. Validation cohort summary.docx]

Supplemental Table 1. The characteristics of breast cancer patients in Validation cohort (total 203 patients), continues features are shown as median (1st to 3rd quantile) and classified features are shown as numbers (percentage). Odd ratio (95% confident interval) and corresponding P values in logistical regression for the events of lymphopenia, the adjusted P values are P values after Bonferroni correction.

| feature | subgroup | median (1st – 3rd Qu) or number (percentage) | | odd ratio (95%CI) | P value | adjusted P value |
| --- | --- | --- | --- | --- | --- | --- |
|  |  | Without  Lymphopenia | With  Lymphopenia |  |  |  |
| event of lymphopenia |  | 99 | 104 |  |  |  |
| baseline white blood cells | | 5.76(4.92-7.34) | 5.19(4.08-6.21) | 0.83(0.71-0.96) | 0.018 | 0.92 |
| baseline hemoglobin | | 120(112-128) | 120(109-126) | 0.99(0.97-1.01) | 0.38 | 1 |
| baseline platelet | | 238(208-283) | 233(199-284) | 1(0.99-1) | 0.35 | 1 |
| baseline neutrophils | | 3.29(2.59-4.63) | 3.22(2.21-4.34) | 0.97(0.83-1.13) | 0.65 | 1 |
| baseline lymphocytes | | 1.82(1.42-2.26) | 1.34(1.12-1.62) | 0.19(0.097-0.34) | <0.001 | <0.001 |
| baseline monocytes | | 0.38(0.29-0.5) | 0.35(0.28-0.46) | 0.66(0.12-3.48) | 0.62 | 1 |
| RT technology | RapidArc | 5(5.44) | 29(30.21) | reference |  |  |
|  | 2D-fields | 58(63.04) | 21(21.87) | 0.062(0.019-0.17) | <0.001 | <0.001 |
|  | 3D-fields | 29(31.52) | 46(47.92) | 0.27(0.086-0.736) | 0.016 | 0.81 |
| RT fields | Trangential breast only | 47(62.67) | 19(26.76) | reference |  |  |
|  | Breast/chest wall with regional lymphatics | 28(37.33) | 52(73.24) | 4.59(2.31-9.45) | <0.001 | 0.001 |
| RT Dose | 40.5Gy/15fx | 86(93.48) | 85(88.54) | reference |  |  |
|  | more than 50Gy/25fx | 6(6.52) | 11(11.46) | 1.85(0.674-5.59) | 0.244 | 1 |
| electron | none | 21(22.83) | 53(55.21) | reference |  |  |
|  | 10Gy/5fx | 63(68.48) | 39(40.63) | 0.25(0.13-0.46) | <0.001 | <0.001 |
|  | 16Gy/8fx | 8(8.69) | 4(4.16) | 0.19(0.048-0.7) | 0.015 | 0.74 |
| mean heart dose | | 2.18(0.4-3.1) | 2.86(0.53-4.07) | 1.3(1.1-1.56) | 0.003 | 0.14 |
| maximum heart dose | | 40.27(4.23-41.71) | 29.34(6.03-42.01) | 1.01(0.99-1.03) | 0.24 | 1 |
| integral dose of the total body | | 3.39(2.98-4.2) | 4.69(2.78-5.06) | 1.93(1.53-2.49) | <0.001 | <0.001 |
| V20 of ipsilateral lung | | 14.75(11.2-19.48) | 22.95(17.25-26.43) | 1.15(1.1-1.21) | <0.001 | <0.001 |
| V5 of ipsilateral lung | | 27.15(21.8-38.58) | 44.85(33.6-59.2) | 1.07(1.05-1.1) | <0.001 | <0.001 |
| mean ipsilateral lung dose | | 7.26(5.88-9.48) | 11.6(8.53-12.93) | 1.41(1.27-1.58) | <0.001 | <0.001 |
| V20 of bilateral lungs | | 6.95(5.6-9.48) | 11.55(8.32-13.6) | 1.29(1.18-1.41) | <0.001 | <0.001 |
| V5 of bilateral lungs | | 13.4(11.03-18.38) | 23.3(16.2-40.92) | 1.09(1.06-1.13) | <0.001 | <0.001 |
| mean bilateral lungs dose | | 3.56(2.99-4.91) | 6.04(4.12-7.86) | 1.64(1.4-1.94) | <0.001 | <0.001 |
| age |  | 45(39-50) | 45(39-52) | 1(0.97-1.03) | 0.89 | 1 |
| family history | without | 46(46.46) | 43(41.35) | reference |  |  |
|  | with | 53(53.54) | 61(58.65) | 1.23(0.71-2.15) | 0.46 | 1 |
| smoking history | without | 55(55.56) | 52(50) | reference |  |  |
|  | with | 0(0) | 2(1.92) | NA | 0.99 | 1 |
|  | unknown | 44(44.44) | 50(48.08) | 1.2(0.69-2.1) | 0.52 | 1 |
| drinking history | without | 55(55.56) | 54(51.92) | reference |  |  |
|  | with | 0(0) | 0(0) | NA |  |  |
|  | unknown | 44(44.44) | 50(48.08) | 1.16(0.67-2.01) | 0.61 | 1 |
| menopausal | premenopausal | 57(69.67) | 54(63.53) | reference |  |  |
|  | perimenopausal | 9(10.85) | 14(16.01) | 1.64(0.67-4.24) | 0.29 | 1 |
|  | postmenopausal | 17(20.48) | 17(20) | 1.06(0.49-2.29) | 0.89 | 1 |
| modified Nstage | 0 | 44(58.67) | 22(30.99) | reference |  |  |
|  | more than 0 | 31(41.33) | 49(69.01) | 3.16(1.62-6.33) | <0.001 | 0.046 |
| modified stage | I | 36(37.89) | 15(15.46) | reference |  |  |
|  | II | 44(46.32) | 41(42.27) | 2.24(1.08-4.77) | 0.032 | 1 |
|  | III | 15(15.79) | 41(42.37) | 6.56(2.89-15.7) | <0.001 | <0.001 |
| tumor sides | tumor side at left | 50(54.35) | 52(54.17) | reference |  |  |
|  | tumor side at right | 42(45.65) | 44(45.83) | 1.01(0.57-1.79) | 0.98 | 1 |
| tumor size | | 2(1.3-2.65) | 2.05(1.5-3) | 1.2(0.99-1.47) | 0.068 | 1 |
| ER | - | 26(26.26) | 31(29.81) | reference |  |  |
|  | + | 73(73.73) | 73(70.19) | 0.84(0.45-1.55) | 0.58 | 1 |
| PR | - | 33(33.33) | 39(37.5) | reference |  |  |
|  | + | 66(66.67) | 65(62.5) | 0.83(0.47-1.48) | 0.54 | 1 |
| HER2 | - | 73(73.74) | 77(74.04) | reference |  |  |
|  | + | 26(26.26) | 27(25.96) | 0.99(0.53-1.85) | 0.96 | 1 |
| IHC | HR+/HER2- | 57(57.58) | 57(54.81) | reference |  |  |
|  | HR-/HER2+ | 9(9.09) | 8(7.69) | 0.89(0.31-2.48) | 0.82 | 1 |
|  | HR+/HER2+ | 19(19.19) | 19(18.27) | 1(0.48-2.09) | 1 | 1 |
|  | HR-/HER2- | 14(14.14) | 20(19.23) | 1.43(0.66-3.15) | 0.34 | 1 |
| Ki67 |  | 30(20-50) | 30(20-50) | 1(0.99-1.01) | 0.75 | 1 |
| surgery regimens | BCT | 58(59.18) | 41(40.2) | reference |  |  |
|  | MRM | 40(40.82) | 61(59.8) | 2.16(1.23-3.82) | 0.008 | 0.38 |
| surgery regimens | SLNB | 52(52.52) | 27(26.47) | reference |  |  |
|  | ALND | 47(47.48) | 75(73.53) | 3.07(1.72-5.61) | <0.001 | 0.009 |
| margin | clear margin | 81(92.05) | 83(93.26) | reference |  |  |
|  | close or positive margin | 7(7.95) | 6(6.74) | 0.84(0.26-2.62) | 0.75 | 1 |
| chemotherapy strategy | none | 13(14.29) | 2(2.08) | reference |  |  |
|  | neoadjuvant | 14(15.38) | 21(21.88) | 9.75(2.25-68.8) | 0.006 | 0.33 |
|  | adjuvant | 60(65.94) | 65(67.71) | 7.04(1.85-46.2) | 0.012 | 0.62 |
|  | neoadjuvant+adjuvant | 4(4.39) | 8(8.33) | 13(2.23-115) | 0.008 | 0.43 |
| chemotherapy regimens | others | 35(35.36) | 38(36.54) | reference |  |  |
|  | taxane | 31(31.31) | 13(12.5) | 0.39(0.17-0.84) | 0.018 | 0.94 |
|  | anthracycline+taxane | 33(33.33) | 53(50.96) | 1.48(0.79-2.8) | 0.23 | 1 |
| anti Her2 therapy | without | 68(75.56) | 77(80.21) | reference |  |  |
|  | with | 22(24.44) | 19(19.79) | 0.76(0.34-1.53) | 0.45 | 1 |
| endocrine therapy | without | 31(31.31) | 36(34.62) | reference |  |  |
|  | with | 68(68.69) | 68(65.38) | 0.86(0.48-1.55) | 0.62 | 1 |
| Abbreviations:  RT: radiation treatment; ER: estrogen receptors; PR: progesterone receptors; IHC: immunohistochemistry; HR: hormone receptor; HER2: human epidermal growth factor receptor 2; BCT: breast-conserving therapy; MRM: modified radical mastectomy; SLNB: Sentinel lymph node biopsy; ALND: axillary lymph node dissection. | | | | | | |
