# Supplementary material for: Potential Determinants for Radiation-Induced Lymphopenia in Patients With Breast Cancer Using Interpretable Machine Learning Approach
Source: Front Immunol. 2022 Jun 21;13:768811. doi: 10.3389/fimmu.2022.768811 (PMC9253393; doi:10.3389/fimmu.2022.768811)
Supplement: Supplementary file 1 [file DataSheet_1.zip › final files/Table S2. the summarized in full XGboost models.docx]

Supplemental Table 2. The gain and frequency indexes of dummy features in the full XGboost models via all iterations. Both gain and frequency indexes are shown as mean, range, 95% confident interval (95%CI) and corresponding *P* values.

| Feature | Gain mean | Gain  (min-max) | Gain  (95%CI) | Gain  *P* value | Frequency mean (%) | Frequency (min-max) (%) | Frequency 95%CI (%) | Frequency *P* value |
| --- | --- | --- | --- | --- | --- | --- | --- | --- |
| baseline lymphocytes | 0.277 | (0.197-0.373) | (0.27-0.284) | <0.001 | 23 | (17.5-29.7) | (22.6-23.5) | <0.001 |
| integral dose of the total body | 0.187 | (0.088-0.299) | (0.178-0.197) | <0.001 | 14.2 | (7.87-20.8) | (13.6-14.8) | <0.001 |
| mean bilateral lungs dose | 0.051 | (0.007-0.199) | (0.045-0.058) | <0.001 | 4.31 | (0.559-13.2) | (3.86-4.76) | <0.001 |
| V5 of bilateral lungs | 0.078 | (0.011-0.201) | (0.07-0.086) | <0.001 | 6.59 | (1.64-11.7) | (6.12-7.07) | <0.001 |
| V5 of ipsilateral lung | 0.068 | (0.012-0.141) | (0.062-0.073) | <0.001 | 5.25 | (1.09-11.6) | (4.86-5.64) | <0.001 |
| mean heart dose | 0.034 | (0.006-0.087) | (0.031-0.037) | <0.001 | 4.85 | (1.08-10.9) | (4.47-5.22) | <0.001 |
| maxim heart dose | 0.036 | (0.007-0.084) | (0.032-0.039) | <0.001 | 5.36 | (1.39-11.3) | (4.88-5.83) | <0.001 |
| baseline white blood cells | 0.043 | (0.007-0.086) | (0.04-0.0465) | <0.001 | 5.13 | (1.13-8.94) | (4.8-5.46) | <0.001 |
| mean ipsilateral lung dose | 0.028 | (0.001-0.086) | (0.025-0.032) | <0.001 | 2.65 | (0.267-7.28) | (2.37-2.94) | <0.001 |
| baseline hemoglobin | 0.028 | (0.005-0.062) | (0.025-0.03) | <0.001 | 4.41 | (1.09-9.43) | (4.03-4.79) | <0.001 |
| chemotherapy regimens: anthracycline+taxane | 0.014 | (0.001-0.077) | (0.011-0.016) | <0.001 | 1.85 | (0.26-7.63) | (1.53-2.16) | <0.001 |
| baseline platelet | 0.023 | (0.002-0.065) | (0.021-0.026) | <0.001 | 3.55 | (0.27-9.04) | (3.2-3.89) | <0.001 |
| baseline monocytes | 0.02 | (0.002-0.067) | (0.017-0.022) | <0.001 | 2.97 | (0.29-7.48) | (2.65-3.3) | <0.001 |
| RT technology: RapidArc | 0.008 | (0.001-0.082) | (0.005-0.011) | <0.001 | 0.82 | (0.26-5.9) | (0.58-1.05) | <0.001 |
| V20 of ipsilateral lung | 0.023 | (0.002-0.089) | (0.019-0.027) | <0.001 | 2.14 | (0.27-5.71) | (1.89-2.38) | <0.001 |
| tumor size | 0.014 | (0.001-0.044) | (0.012-0.016) | <0.001 | 2.33 | (0.28-6.93) | (2.01-2.65) | <0.001 |
| chemotherapy regimens: taxane | 0.011 | (0.001-0.047) | (0.009-0.013) | <0.001 | 1.66 | (0.27-5.88) | (1.41-1.91) | <0.001 |
| Ki67 | 0.01 | (0.001-0.041) | (0.008-0.012) | <0.001 | 1.64 | (0.27-6.47) | (1.37-1.91) | <0.001 |
| baseline neutrophils | 0.017 | (0.001-0.042) | (0.015-0.019) | <0.001 | 2.46 | (0.28-5.28) | (2.21-2.7) | <0.001 |
| age | 0.008 | (0.001-0.028) | (0.006-0.009) | <0.001 | 1.3 | (0.26-4.26) | (1.1-1.51) | <0.001 |
| without HER2 | 0.006 | (0.001-0.024) | (0.005-0.008) | <0.001 | 1 | (0.25-3.07) | (0.81-1.18) | <0.001 |
| V20 of bilateral lungs | 0.008 | (0.001-0.038) | (0.007-0.01) | <0.001 | 1.03 | (0.25-3.45) | (0.90-1.16) | <0.001 |
| with HER2 | 0.003 | (0.001-0.016) | (0.002-0.004) | <0.001 | 0.51 | (0.25-1.92) | (0.4-0.62) | <0.001 |
| RT technology: 3D-fields | 0.003 | (0.001-0.011) | (0.003-0.004) | <0.001 | 0.54 | (0.25-2.25) | (0.44-0.64) | <0.001 |
| HR-/HER2+ | 0.003 | (0.001-0.011) | (0.001-0.004) | <0.001 | 0.49 | (0.26-2.1) | (0.24-0.74) | <0.001 |
| HR+/HER2+ | 0.004 | (0.001-0.012) | (0.003-0.006) | <0.001 | 0.72 | (0.26-2.01) | (0.51-0.93) | <0.001 |
| electron: 10Gy/5fx | 0.003 | (0.001-0.01) | (0.002-0.004) | <0.001 | 0.54 | (0.25-2.13) | (0.4-0.67) | <0.001 |
| HR+/HER2- | 0.003 | (0.001-0.012) | (0.002-0.004) | <0.001 | 0.54 | (0.25-1.74) | (0.41-0.64) | <0.001 |
| neoadjuvant chemotherapy | 0.004 | (0.001-0.012) | (0.003-0.005) | <0.001 | 0.64 | (0.26-1.68) | (0.48-0.79) | <0.001 |
| with family history | 0.003 | (0.001-0.01) | (0.002-0.004) | <0.001 | 0.49 | (0.26-1.66) | (0.31-0.66) | <0.001 |
| modified stage II | 0.003 | (0.001-0.01) | (0.003-0.004) | <0.001 | 0.52 | (0.25-1.4) | (0.43-0.62) | <0.001 |
| BCT | 0.002 | (0.001-0.009) | (0.002-0.003) | <0.001 | 0.38 | (0.25-1.39) | (0.24-0.52) | <0.001 |
| without endocrine therapy | 0.002 | (0.001-0.008) | (0.002-0.003) | <0.001 | 0.39 | (0.25-1.29) | (0.29-0.49) | <0.001 |
| modified stage I | 0.003 | (0.001-0.01) | (0.001-0.005) | 0.0106 | 0.38 | (0.25-0.83) | (0.22-0.55) | <0.001 |
| modified stage III | 0.003 | (0.001-0.01) | (0.002-0.004) | <0.001 | 0.41 | (0.27-0.94) | (0.29-0.52) | <0.001 |
| SLNB | 0.003 | (0.001-0.014) | (0.001-0.004) | 0.0136 | 0.32 | (0.26-0.56) | (0.25-0.38) | <0.001 |
| ALND | 0.004 | (0.001-0.012) | (0-0.008) | 0.039 | 0.32 | (0.26-0.55) | (0.22-0.41) | <0.001 |
| none chemotherapy | 0.003 | (0.001-0.006) | (0.001-0.005) | 0.011 | 0.41 | (0.27-1.06) | (0.07-0.74) | 0.026 |
| without PR | 0.002 | (0.001-0.006) | (0.002-0.003) | <0.001 | 0.35 | (0.26-0.84) | (0.28-0.42) | <0.001 |
| chemotherapy regimens: others | 0.003 | (0.001-0.005) | (-0.001-0.006) | 0.081 | 0.42 | (0.27-0.85) | (-0.04-0.88) | 0.063 |
| with antiHER2 therapy | 0.002 | (0.001-0.004) | (0.001-0.003) | <0.001 | 0.43 | (0.27-1.07) | (0.15-0.71) | 0.009 |
| without smoking history | 0.002 | (0.001-0.005) | (0.001-0.003) | <0.001 | 0.37 | (0.26-0.8) | (0.28-0.46) | <0.001 |
| without ER | 0.002 | (0.001-0.005) | (0.002-0.003) | <0.001 | 0.36 | (0.25-0.84) | (0.28-0.43) | <0.001 |
| unknown smoking history | 0.002 | (0.001-0.005) | (0.001-0.003) | <0.001 | 0.31 | (0.26-0.76) | (0.23-0.39) | <0.001 |
| electron: none | 0.002 | (0.001-0.005) | (0.001-0.003) | 0.002 | 0.35 | (0.25-0.86) | (0.18-0.52) | 0.002 |
| premenopausal | 0.002 | (0.001-0.004) | (0.001-0.003) | <0.001 | 0.38 | (0.26-0.89) | (0.23-0.54) | <0.001 |
| without drinking history | 0.002 | (0.001-0.006) | (0.001-0.003) | <0.001 | 0.33 | (0.26-0.62) | (0.26-0.4) | <0.001 |
| tumor side at left | 0.003 | (0.001-0.006) | (0.002-0.003) | <0.001 | 0.36 | (0.25-0.58) | (0.3-0.4) | <0.001 |
| modified N stage 0 | 0.003 | (0.001-0.01) | (0-0.007) | 0.084 | 0.27 | (0.26-0.28) | (0.26-0.28) | <0.001 |
| RT technology: 2D-fields | 0.005 | (0.002-0.011) | (0-0.009) | 0.04 | 0.27 | (0.26-0.28) | (0.26-0.28) | <0.001 |
| without antiHER2 therapy | 0.002 | (0.001-0.005) | (0.002-0.003) | <0.001 | 0.37 | (0.26-0.56) | (0.31-0.43) | <0.001 |
| postmenopausal | 0.003 | (0.001-0.005) | (0.002-0.003) | <0.001 | 0.39 | (0.26-0.55) | (0.28-0.5) | <0.001 |
| adjuvant chemotherapy | 0.002 | (0.001-0.004) | (0.002-0.003) | <0.001 | 0.32 | (0.26-0.6) | (0.27-0.36) | <0.001 |
| without family history | 0.002 | (0.001-0.004) | (0.001-0.003) | 0.002 | 0.33 | (0.27-0.57) | (0.23-0.42) | <0.001 |
| with ER | 0.002 | (0.001-0.004) | (0.002-0.003) | <0.001 | 0.33 | (0.26-0.6) | (0.24-0.42) | <0.001 |
| with PR | 0.002 | (0.001-0.004) | (0.001-0.002) | <0.001 | 0.32 | (0.25-0.54) | (0.25-0.38) | <0.001 |
| with endocrine therapy | 0.002 | (0.001-0.003) | (0.001-0.002) | <0.001 | 0.3 | (0.25-0.52) | (0.25-0.34) | <0.001 |
| MRM | 0.002 | (0.002-0.003) | (0.001-0.003) | 0.016 | 0.37 | (0.27-0.56) | (-0.04-0.77) | 0.059 |
| tumor side at right | 0.002 | (0.001-0.003) | (0.002-0.002) | <0.001 | 0.27 | (0.25-0.3) | (0.26-0.28) | <0.001 |
| unknown drinking history | 0.001 | (0.001-0.002) | (0.001-0.002) | <0.001 | 0.28 | (0.25-0.31) | (0.26-0.3) | <0.001 |
| HR-/HER2- | 0.001 | (0.001-0.002) | (0-0.003) | 0.049 | 0.28 | (0.27-0.29) | (0.25-0.3) | <0.001 |
| RT fields: Trangential breast only | 0 | (0-0) | (0-0) | 0 | 0 | (0-0) | (0-0) | 0 |
| modified N stage more than 0 | 0 | (0-0) | (0-0) | 0 | 0 | (0-0) | (0-0) | 0 |
| RT fields: Breast/chest wall with regional lymphatics | 0 | (0-0) | (0-0) | 1 | 0 | (0-0) | (0-0) | 1 |
| RT Dose: 40.5Gy/15fx | 0 | (0-0) | (0-0) | 1 | 0 | (0-0) | (0-0) | 1 |
| RT Dose: more than 50Gy/25fx | 0 | (0-0) | (0-0) | 1 | 0 | (0-0) | (0-0) | 1 |
| electron: 16Gy/8fx | 0 | (0-0) | (0-0) | 1 | 0 | (0-0) | (0-0) | 1 |
| with smoking history | 0 | (0-0) | (0-0) | 1 | 0 | (0-0) | (0-0) | 1 |
| with drinking history | 0 | (0-0) | (0-0) | 1 | 0 | (0-0) | (0-0) | 1 |
| perimenopausal | 0 | (0-0) | (0-0) | 1 | 0 | (0-0) | (0-0) | 1 |
| clear margin | 0 | (0-0) | (0-0) | 1 | 0 | (0-0) | (0-0) | 1 |
| close or positive margin | 0 | (0-0) | (0-0) | 1 | 0 | (0-0) | (0-0) | 1 |
| neoadjuvant+adjuvant chemotherapy | 0 | (0-0) | (0-0) | 1 | 0 | (0-0) | (0-0) | 1 |
| Abbreviations:  RT: radiation treatment; ER: estrogen receptors; PR: progesterone receptors; IHC: immunohistochemistry; HR: hormone receptor; HER2: human epidermal growth factor receptor 2; BCT: breast-conserving therapy; MRM: modified radical mastectomy; SLNB: Sentinel lymph node biopsy; ALND: axillary lymph node dissection. | | | | | | | | |
