# Supplementary material for: Potential Determinants for Radiation-Induced Lymphopenia in Patients With Breast Cancer Using Interpretable Machine Learning Approach
Source: Front Immunol. 2022 Jun 21;13:768811. doi: 10.3389/fimmu.2022.768811 (PMC9253393; doi:10.3389/fimmu.2022.768811)
Supplement: Supplementary file 1 [file DataSheet_1.zip › final files/Table S3. the summarized in full Lasso regressions.docx]

Supplemental Table 2. The coefficients of dummy features in the full Lasso regressions via all iterations. The coefficients are shown as mean, range, 95% confident interval (95%CI), corresponding *P* values and occurrence frequencies across all iterations.

| Feature | Coefficient  mean | Coefficient  (min-max) | Coefficient  (95%CI) | *P* value | Frequency (%) |
| --- | --- | --- | --- | --- | --- |
| baseline lymphocytes | -0.902 | (-1.7--0.283) | (-0.953--0.852) | <0.001 | 100 |
| integral dose of the total body | 0.487 | (0.008-1.22) | (0.436-0.538) | <0.001 | 96 |
| V5 of ipsilateral lung | 0.349 | (0.002-0.888) | (0.31-0.388) | <0.001 | 84 |
| V5 of bilateral lungs | 0.282 | (0.027-0.714) | (0.24-0.323) | <0.001 | 61 |
| chemotherapy regimens: taxane | -0.334 | (-1.11--0.012) | (-0.389--0.28) | <0.001 | 60 |
| RT technology: RapidArc | 0.759 | (0.001-3.75) | (0.575-0.943) | <0.001 | 54 |
| chemotherapy regimens: anthracycline+taxane | 0.238 | (0.001-0.755) | (0.184-0.291) | <0.001 | 43 |
| baseline platelet | -0.104 | (-0.334--0.005) | (-0.136--0.073) | <0.001 | 34 |
| neoadjuvant chemotherapy | 0.321 | (0.002-0.776) | (0.248-0.395) | <0.001 | 32 |
| without HER2 | 0.29 | (0.007-1.64) | (0.187-0.394) | <0.001 | 31 |
| electron: 16Gy/8fx | -0.273 | (-1.29--0.001) | (-0.387--0.159) | <0.001 | 29 |
| clear margin | 0.39 | (0.003-1.83) | (0.218-0.562) | <0.001 | 23 |
| Ki67 | -0.092 | (-0.366-0.03) | (-0.134--0.05) | <0.001 | 22 |
| with smoking history | -0.6 | (-2.25--0.011) | (-0.877--0.323) | <0.001 | 20 |
| with HER2 | -0.003 | (-0.043-0) | (-0.007-0.002) | 0.276 | 19 |
| baseline hemoglobin | 0.124 | (0.001-0.342) | (0.077-0.172) | <0.001 | 15 |
| mean bilateral lungs dose | 0.171 | (0-0.618) | (0.082-0.259) | 0.001 | 15 |
| HR+/HER2+ | -0.245 | (-0.659--0.04) | (-0.35--0.141) | <0.001 | 14 |
| baseline monocytes | -0.063 | (-0.178--0.004) | (-0.093--0.033) | <0.001 | 13 |
| tumor side at left | -0.103 | (-0.313-0.05) | (-0.167--0.039) | 0.004 | 13 |
| neoadjuvant+adjuvant chemotherapy | -0.225 | (-1.93-0.571) | (-0.703-0.254) | 0.324 | 12 |
| tumor side at right | 0 | (0-0) | (0-0) | 0.209 | 10 |
| baseline white blood cells | -0.069 | (-0.121--0.015) | (-0.102--0.035) | 0.001 | 9 |
| RT Dose: 40.5Gy/15fx | -0.456 | (-1.37--0.012) | (-0.818--0.095) | 0.02 | 8 |
| perimenopausal | -0.281 | (-0.577--0.024) | (-0.473--0.089) | 0.01 | 8 |
| close or positive margin | -0.005 | (-0.018-0) | (-0.012-0.002) | 0.17 | 8 |
| with family history | 0.047 | (-0.157-0.204) | (-0.067-0.161) | 0.349 | 7 |
| HR+/HER2- | 0.159 | (0.006-0.416) | (0.029-0.288) | 0.024 | 7 |
| HR-/HER2+ | -0.244 | (-0.765-0.189) | (-0.58-0.092) | 0.125 | 7 |
| mean ipsilateral lung dose | 0.153 | (0.077-0.228) | (0.087-0.218) | 0.002 | 6 |
| modified stage II | -0.056 | (-0.406-0.335) | (-0.324-0.213) | 0.618 | 6 |
| none chemotherapy | -0.311 | (-0.575--0.007) | (-0.512--0.11) | 0.011 | 6 |
| RT Dose: more than 50Gy/25fx | 0.003 | (9-0.014) | (-0.005-0.011) | 0.374 | 5 |
| electron: 10Gy/5fx | 0.276 | (0.003-0.905) | (-0.184-0.735) | 0.171 | 5 |
| V20 of bilateral lungs | 0.118 | (0.009-0.39) | (-0.173-0.408) | 0.287 | 4 |
| age | 0.016 | (-0.011-0.071) | (-0.043-0.076) | 0.446 | 4 |
| without family history | 0 | (0-0) | (0-0.001) | 0.391 | 4 |
| postmenopausal | -0.151 | (-0.215--0.06) | (-0.254--0.047) | 0.019 | 4 |
| modified stage III | 0.019 | (0.002-0.056) | (-0.021-0.06) | 0.22 | 4 |
| tumor size | 0 | (-0.042-0.037) | (-0.052-0.051) | 0.992 | 4 |
| without ER | 0.097 | (-0.292-0.937) | (-0.826-1.02) | 0.759 | 4 |
| with ER | 0 | (0-0) | (0-0) | 0.429 | 4 |
| BCT | -0.3 | (-0.592--0.032) | (-0.76-0.161) | 0.13 | 4 |
| MRM | 0 | (0-0) | (0-0) | 0.323 | 4 |
| SLNB | 0.15 | (-0.092-0.408) | (-0.224-0.524) | 0.291 | 4 |
| ALND | 0 | (0-0) | (0-0) | 0.391 | 4 |
| without endocrine therapy | -0.288 | (-0.673--0.107) | (-0.713-0.138) | 0.12 | 4 |
| baseline neutrophils | 0.06 | (0.021-0.105) | (-0.045-0.166) | 0.132 | 3 |
| without drinking history | -0.796 | (-2.14--0.06) | (-3.68-2.09) | 0.357 | 3 |
| premenopausal | 0.187 | (0.029-0.406) | (-0.298-0.673) | 0.239 | 3 |
| without PR | -0.195 | (-0.335--0.069) | (-0.527-0.136) | 0.126 | 3 |
| adjuvant chemotherapy | -0.105 | (-0.239-0.005) | (-0.412-0.201) | 0.278 | 3 |
| with endocrine therapy | 0 | (0-0) | (0-0) | 0.245 | 3 |
| RT technology: 2D-fields | 0 | (0-0) | (0-0) | 1 | 0 |
| RT technology: 3D-fields | 0 | (0-0) | (0-0) | 1 | 0 |
| RT fields: Trangential breast only | 0 | (0-0) | (0-0) | 1 | 0 |
| RT fields: Breast/chest wall with regional lymphatics | 0 | (0-0) | (0-0) | 1 | 0 |
| electron: none | 0 | (0-0) | (0-0) | 1 | 0 |
| mean heart dose | 0 | (0-0) | (0-0) | 1 | 0 |
| maxim heart dose | 0 | (0-0) | (0-0) | 1 | 0 |
| V20 of ipsilateral lung | 0 | (0-0) | (0-0) | 1 | 0 |
| without smoking history | 0 | (0-0) | (0-0) | 1 | 0 |
| unknown smoking history | 0 | (0-0) | (0-0) | 1 | 0 |
| with drinking history | 0 | (0-0) | (0-0) | 1 | 0 |
| unknown drinking history | 0 | (0-0) | (0-0) | 1 | 0 |
| modified N stage 0 | 0 | (0-0) | (0-0) | 1 | 0 |
| modified N stage more than 0 | 0 | (0-0) | (0-0) | 1 | 0 |
| modified stage I | 0 | (0-0) | (0-0) | 1 | 0 |
| with PR | 0 | (0-0) | (0-0) | 1 | 0 |
| HR-/HER2- | 0 | (0-0) | (0-0) | 1 | 0 |
| chemotherapy regimens: others | 0 | (0-0) | (0-0) | 1 | 0 |
| without antiHER2 therapy | 0 | (0-0) | (0-0) | 1 | 0 |
| with antiHER2 therapy | 0 | (0-0) | (0-0) | 1 | 0 |
| Abbreviations:  RT: radiation treatment; ER: estrogen receptors; PR: progesterone receptors; IHC: immunohistochemistry; HR: hormone receptor; HER2: human epidermal growth factor receptor 2; BCT: breast-conserving therapy; MRM: modified radical mastectomy; SLNB: Sentinel lymph node biopsy; ALND: axillary lymph node dissection. | | | | | |
